# Biological Activities of Constituents from *Rosa roxburghii* and Their Mechanisms Based on Network Pharmacology and Biological Verification

**DOI:** 10.3390/ijms26031353

**Published:** 2025-02-05

**Authors:** Li-Juan Xiang, Shuang Zhang, Ming-Liang Luo, Xing-Xiang Long, Ying Zhou, Xin Yin

**Affiliations:** College of Pharmacy, Guizhou University of Traditional Chinese Medicine, Guiyang 550025, China; xiang1820848@163.com (L.-J.X.); rocmsw@163.com (S.Z.); 15085565499@163.com (M.-L.L.); l18786574663@163.com (X.-X.L.)

**Keywords:** *Rosa roxburghii* tratt roots, chemical constituents, anti-inflammatory, network pharmacological, molecular docking

## Abstract

*Rosa roxburghii* Tratt is widely cultivated in southwestern areas of China for a range of purposes, including food and medicine. To enhance its application value, one previously undescribed compound (**1**) and six known compounds (**2**–**7**) were isolated from the roots of *R. roxburghii*. The structures of compounds (**1**–**7**) were determined through NMR, HR-MS, and CD experiments, and by comparison of their spectroscopic data with values from the literature. Roxbubenzoate A (**1**) contained a benzoyl glucuronosyl glycerol scaffold featuring a rare *α*-glucuronosyl linkage. Seven compounds (**1**–**7**) were tested for their anti-inflammatory, *α*-glucosidase-inhibitory, and radical-scavenging activities. Compound **3** showed a significant inhibitory effect on the release of NO in LPS-induced RAW264.7 macrophages, with an IC_50_ value of 7.8 ± 0.2 μM. Compounds **2**, **4**, and **7** exhibited moderate inhibitory activity against *α*-glucosidase, with inhibition rates of 50.1%, 46.7%, and 41.1% at a concentration of 200 μM, respectively. Compounds **1**, **2**, and **6** exhibited moderate ABTS radical-scavenging activity, with IC_50_ values of 107.0 ± 1.1, 142.6 ± 0.8, and 128.3 ± 1.2 μM, respectively. The network pharmacological analysis and molecular docking results suggested that **3** may be able to treat inflammation by binding TNF-*α* and IL-6 targets. Finally, the expression of IL-6 and TNF-*α* in LPS-induced RAW264.7 macrophages was detected through ELISA, and **3** showed a strong inhibitory effect on IL-6 release. These findings offer a novel perspective on the development of rich medicinal plant resources from *R. roxburghii* roots.

## 1. Introduction

*Rosa roxburghii* Tratt is a well-established and extensively researched species within the *Rosa* genus and plays a substantial role in the fields of dietary supplements and medicine [1]. The whole of the *R. roxburghii* plant is edible (including fruits, leaves, and roots) and it has traditionally been used in Chinese folk medicine [2]. In recent years, some food products have been developed from *R. roxburghii* fruits, such as tea, vinegar, jam, yogurt, preserved fruit, and cake [3]. *R. roxburghii* fruits exhibit diverse pharmacological activities, including lowering blood lipids and blood sugar and demonstrating anti-atherosclerosis and anti-tumor effects [4,5,6]. Chemical research has indicated that *R. roxburghii* fruits primarily contain flavonoids, polysaccharides, and triterpenoids [4,7,8]. Furthermore, the leaves and roots of *R. roxburghii* are commonly used as raw materials for making tea and wine and in cooking in various communities in China [9]. However, the chemical composition of *R. roxburghii* roots has not been extensively investigated, and only polyphenols, flavonoids, and organic acids have been isolated so far [10,11].

**Figure 1:**
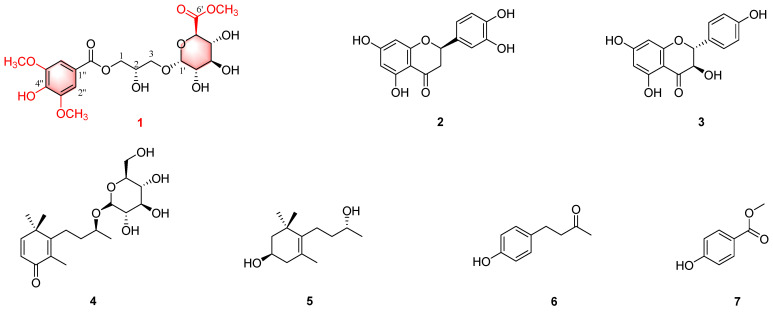
Chemical structures of compounds **1**–**7**.

The diverse array of biological activities exhibited by *R. roxburghii* suggest its considerable potential for medicinal exploration. Type 2 diabetes mellitus (T2DM) is a common chronic metabolic disease that can lead to complications that reduce quality of life and increase mortality. Oxidative stress and inflammation play a major role in the pathogenesis of T2DM, which is currently considered to be an inflammatory and oxidative stress disease [12,13]. In addition, α-glucosidase is an enzyme that breaks down carbohydrates to release glucose, and the inhibitors of α-glucosidase are considered useful drug candidates for T2DM [6]. Discovering natural products with anti-inflammatory, antioxidant, and α-glucosidase-inhibitory activities from plants is an important approach to exploring candidate drugs for T2DM. To enhance the application value of *R. roxburghii*, we conducted a chemical investigation of its roots, resulting in the isolation of one new compound (**1**) and six previously identified compounds (**2**–**7**) (Figure 1). Additionally, the bioactivities of these isolated compounds were evaluated, including anti-inflammatory, *α*-glucosidase-inhibitory and radical-scavenging activities. Details of the structural elucidation, isolation, and biological activity evaluation of **1**–**7** are described herein.

## 2. Results and Discussion

### 2.1. Structural Elucidation

Compound **1** was a pale-yellow oily substance, and the molecular formula can be elucidated as C_19_H_26_O_13_ based on the [M + Na]^+^ peak at *m*/*z* 485.1266 in the HR-ESI-MS spectrum (calcd. for C_19_H_26_O_13_Na^+^, *m*/*z* 485.1265, five degrees of unsaturation) (Figure S7). The IR spectrum indicated the presence of hydroxyl (3420 cm^−1^) and aromatic ring (1547 cm^−1^) functionalities (Figure S11), and the UV spectrum also showed the presence of aromatic rings (277.0 nm) (Figure S10). The ^1^H-NMR (Figure S1) of **1** displayed signals for two aromatic protons, *δ*_H_ 7.37 (2H, s, H-2″, 6″), an anomeric proton, *δ*_H_ 4.89 (1H, d, *J* = 3.7 Hz, H-1′), and three groups of methoxy protons, *δ*_H_ 3.66 (3H, s, H-6′) and 3.90 (6H, s, H-3″, 5″) (Table 1). In ^13^C-NMR (Figure S2), 18 carbon signals are shown (Table 1): the carbon signals of the substituted benzene ring, *δ*_C_ 121.2 (C-1″), 142.0 (C-4″), 108.3 (C-2″, 6″), and 148.9 (C-3″, 5″), 2 carbonyl carbons, *δ*_C_ 171.9 (C-6′) and 167.9 (C-7″), 1 anomeric carbon, *δ*_C_ 101.2 (C-1′), and 3 methoxy carbons, *δ*_C_ 56.9 (C-3″, 5″) and 52.7 (C-6′). Combined with DEPT-135 spectra (Figure S3), two methylene carbon signals were observed at *δ*_C_ 66.6 (C-1) and 70.6 (C-3).

**Figure 2:**
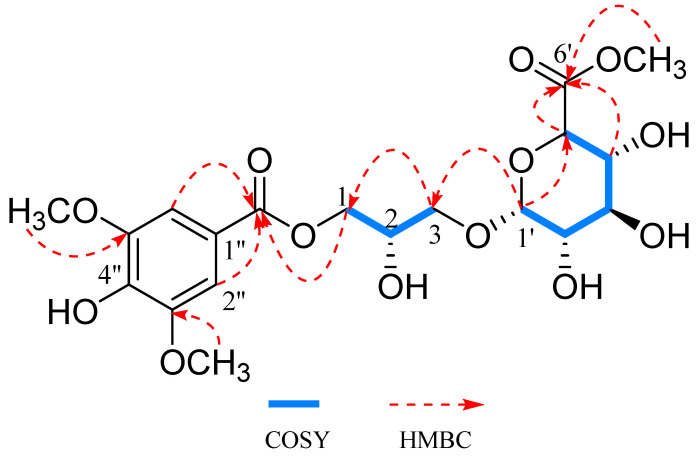
Key HMBC and ^1^H-^1^H COSY correlations of compound **1**.

The ^1^H and ^13^C NMR data of **1** (Table 1) were similar to those of 1-*α*-D-glucuronyl-3-(4-hydroxy)-3,5-dimethoxybenzoyl glycerol, which was obtained previously from the *Schiekia orinocensiss* [14], with the main difference being the presence of an additional methoxy in **1**, as shown by the proton at *δ*_H_ 3.63 (3H, s, 6′-OCH_3_) and the carbon signal at *δ*_C_ 52.7 (6′-OCH_3_) (Figures S4 and S6). The attachment of the methoxy group to C-6′ of **1** was confirmed by the HMBC correlation observed between *δ*_H_ 3.63 (3H, s, 6′-OCH_3_) and *δ*_C_ 171.9 (C-6′) (Figure 2 and Figure S5). The *α*-glucuronosyl linkage in **1** was deduced on the basis of the small *J* value of H-1′ (*J* = 3.7 Hz). The absolute configuration of glucose was identified as D-glucose by a comparison of the specific optical rotation value of its hydrolyzed derivatives.

CD spectrum analyses were used to assign the absolute configuration of **1**. In the CD spectrum (Figure S9), both a positive Cotton effect at 208 nm (Δ*ε* + 1.57) and 275 nm (Δ*ε* + 0.28) and a negative Cotton effect at 231 nm (Δ*ε* − 0.38) indicated that **1** had a 2*S* configuration [15]. Therefore, the structure of **1** was established, and it was informally named roxbubenzoate A.

Additionally, six known compounds, including (-)-eriodictyol (**2**) [16], aromadendrin (**3**) [17], linarionoside A (**4**) [18], (9*S*)-O-*β*-D-glucopyranosyl-2,5-megastigmen-4-one (**5**) [19], (1*R*)-4-[(3*R*)-3-hydroxybutyl]-3,5,5-trimethylcyclohex-3-en-1-ol (**6**) [20], and 4-(1-Methoxyethenyl)phenol (**7**) [21], were identified, and their structures were determined through spectral analysis and comparison with the literature data (Table S1 and Figures S12–S23).

### 2.2. Cell Viability of RAW264.7 Cells

The cytotoxicity of compounds (**1**–**6**) against RAW264.7 cells was determined using the MTT method to obtain their safe concentrations for anti-inflammatory activity. It can be seen from Table S2 in the Supporting Information that compounds (**1**–**6**) had no effect on the viability of RAW264.7 cells at a concentration of 50 μM. Based on this result, a concentration of 50 μM was selected for subsequent anti-inflammatory activity studies.

### 2.3. Inhibition of NO Production Induced by LPS in RAW264.7 Cells

**Figure 3:**
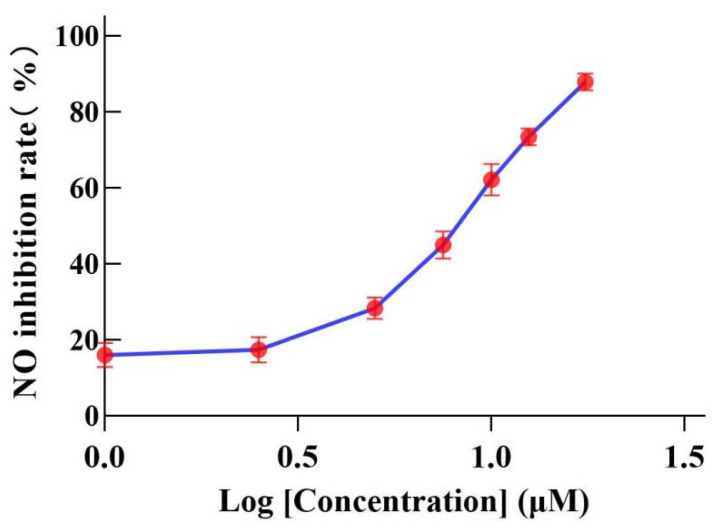
NO inhibitory activities of **3** in the RAW264.7 cell line.

A non-cytotoxic concentration was selected to determine the effects of compounds (**1**–**6**) on the expression of NO in the supernatant of LPS-induced RAW264.7 cells. The experimental results show that **3** has a significant inhibitory effect on NO production, with an IC_50_ of 7.83 ± 2.0 μM, and other compounds were weakly active or inactive (Table S2, Figure 3). Pyrrolidine dithiocarbamate (PDTC) was used as a positive control with an IC_50_ of 3.13 ± 0.3 μM.

### 2.4. α-Glucosidase-Inhibitory Activity

**Figure 4:**
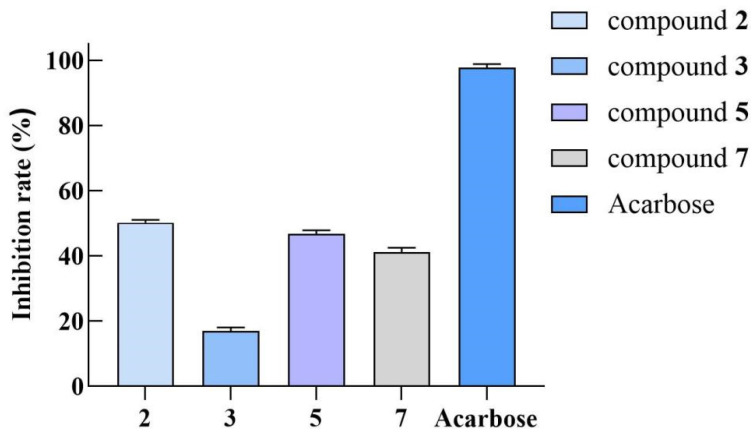
Inhibition rate of compounds (**2**, **3**, **5**, and **7**) and acarbose on *α*-glucosidase at a concentration of 200 μM.

**Figure 5:**
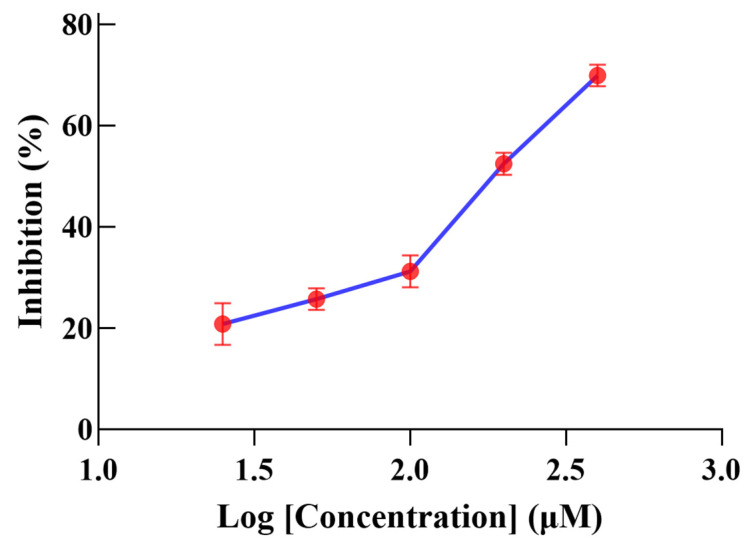
Inhibition activity of **2** against *α*-glucosidase in vitro.

Isolated compounds (**1–7**) were evaluated in vitro for *α*-glucosidase-inhibitory activity at a concentration of 200 μM; **2**, **4**, and **7** exhibited moderate inhibitory activity (inhibition rates: 50.1%, 46.7%, and 41.1%) (Figure 4). Active compound **2** was further tested for *α*-glucosidase-inhibitory activity at different concentrations, and the IC_50_ value was found to be 182.4 ± 0.9 μM (Figure 5).

### 2.5. DPPH and ABTS Free Radical-Scavenging Activity

**Figure 6:**
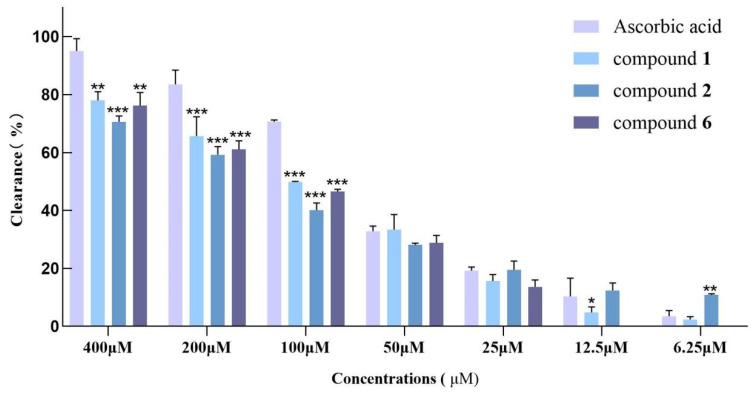
Scavenging ability of compounds **1**, **2**, and **6** on ABTS free radicals. Comparison with ascorbic acid; *** *p* < 0.001, ** *p* < 0.01, * *p* < 0.05.

The antioxidant capacity of compounds (**1**–**7**) was investigated using DPPH and ABTS radical-scavenging assays. Three compounds (**1**, **2**, and **6**) exhibited moderate ABTS radical-scavenging activity, and the IC_50_ values were calculated to be 107.0 ± 1.1, 142.6 ± 0.8, and 128.3 ± 1.2 μM, respectively (Figure 6). Compounds **9** and **10** showed 26.7% and 19.2% scavenging of DPPH radicals at a concentration of 400 μM, respectively. Ascorbic acid was used as a positive control with an IC_50_ of 62.8 ± 2.2 μM.

### 2.6. Network Pharmacology Predicts the Potential Anti-Inflammatory Pathways of Compound ***3***

**Figure 7:**
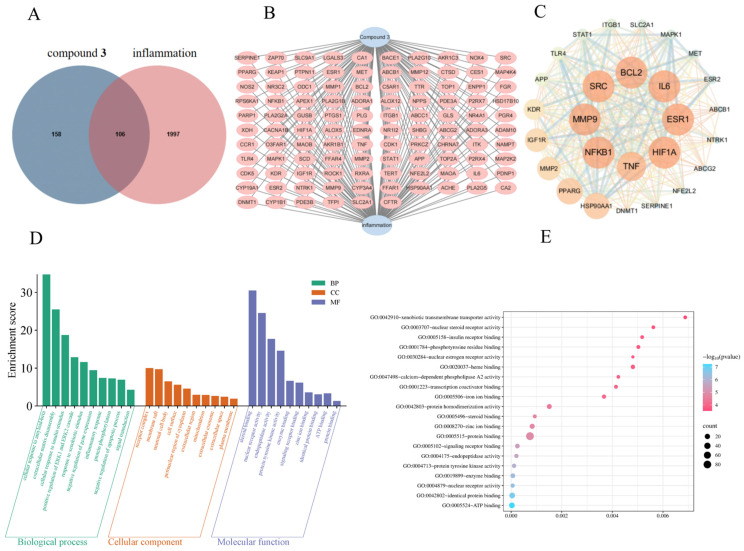
Analysis of the anti-inflammatory action targets of compound **3**. (**A**,**B**) The intersection between compound targets and disease targets. (**C**) Core targets at the intersection of compounds and diseases. Enrichment analysis of core targets: GO function (**D**) and KEGG pathway (**E**).

Based on the results of the three activity assessments, compound **3** exhibited the most significant anti-inflammatory effects. In order to further analyze the mechanism of **3** in the treatment of inflammation, this study conducted further exploration using network pharmacology and a structural virtual screening strategy. Compound **3** was a predicted component-related target, and according to the intersection of the Venn diagram, there were a total of 106 overlapping targets (Figure 7). The protein–protein interaction (PPI) network analyzed in combination with Cytoscape 3.10.0 software yielded 27 core targets (Figure 7). Nodes with higher degrees are presented in darker colors and larger sizes, indicating their closer correlation with **3**. These key genes are TNF, IL-6, BCL2, ESR1, SRC, MMP9, HIF1A, and NFKB1, which may play a crucial role in the anti-inflammatory effect of **3**. In addition, the GO (Gene Ontology) included BPs (Biological Processes), CCs (Cellular Components), and MF (Molecular Function), and the top five items in each category were selected for bar charts (Figure 7). As shown in Figure 7, the BP category mostly involves transcription initiation from cellular response to amyloid-beta, extracellular matrix disassembly, cellular response to insulin stimulus, etc. The CC category mainly focuses on receptor complex, membrane raft, neuronal cell body, etc. The MF category is mostly engaged with steroid binding, nuclear receptor activity, endopeptidase activity, etc. The KEGG results show that the majority of these targets were enriched in ATP binding (n = 27), identical protein binding (n = 28), enzyme binding (n = 13), nuclear receptor activity (n = 7), protein tyrosine kinase activity (n = 8), etc. (Figure 7).

### 2.7. Molecular Docking Analysis

**Figure 8:**
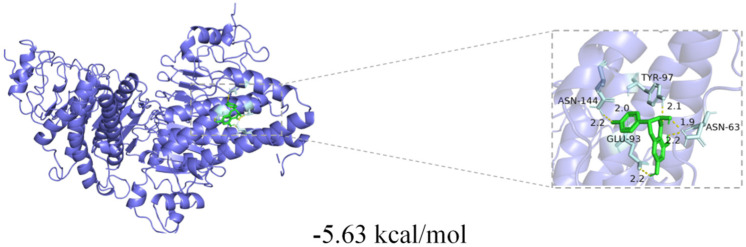
Visualization of IL-6 docking results.

Inflammation is a defensive response of the body to injury or infection. TNF-*α* can act on various inflammatory cells and activate them. When TNF-*α* binds to the corresponding receptors on the surface of inflammatory cells, inflammatory cells are activated, triggering a series of cascades of intracellular signaling pathways and eventually leading to a significant increase in the expression of IL-6 and so on. IL-6 can then continue to act on other cells [22]. Based on the conclusions drawn from the above network pharmacology, two core targets (TNF-*α*, IL-6) were selected for molecular docking, and the results indicate that **3** has strong binding activity, primarily with the IL-6 protein. The visualization of the docking results is shown in Figure 8; **3** had the best docking energy with the IL-6 protein (−5.63 kcal/mol).

### 2.8. The Effect of Compound ***3*** on the Expression of TNF-α and IL-6

**Figure 9:**
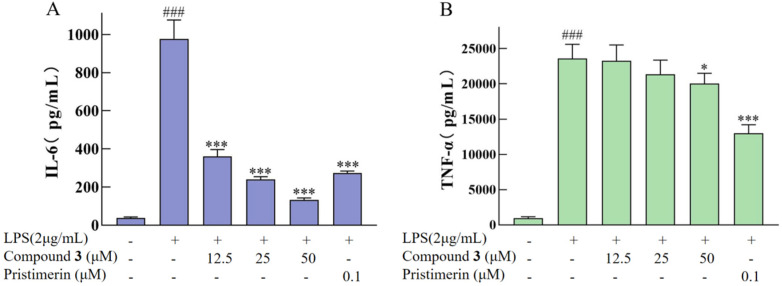
Effects of compound **3** on the expression of IL-6 and TNF-*α* in LPS-stimulated RAW264.7 cells. The expression of IL-6 for **3** (**A**). The expression of TNF-*α* for **3** (**B**). Pristimerin (0.1 μM) was used as a positive control; ### indicates *p* < 0.001 compared with the normal group; * indicates *p* < 0.05; and *** indicates *p* < 0.001 compared with the LPS model group.

TNF-*α* is a key inflammatory factor released early in the inflammatory response, and IL-6 is another important pro-inflammatory factor that can interact with TNF-*α* to further amplify the inflammatory response [23]. ELISA experiments were used to test the regulatory effect of **3** on the expression levels of inflammatory factors (TNF-*α* and IL-6) induced by LPS in the RAW264.7 cell line. The results indicate that the cytokine levels of IL-6 in LPS-stimulated RAW264.7 cells were dose-dependently suppressed by **3**, and compound **3** showed significant inhibitory activity on the expression of IL-6 at the lowest concentration of 12.5 μM in a dose-dependent manner (Figure 9). Compound **3** also showed significant inhibitory activity against TNF-*α* expression at a concentration of 50 μM (Figure 9).

## 3. Materials and Methods

### 3.1. Plant Material

The roots of *R. roxburghii* were collected in Guizhou Province, China, in August 2023, and identified by Professor Shenghua Wei. A voucher specimen (no. 20230809001) was deposited at the College of Pharmacy, Guizhou University of Traditional Chinese Medicine, Guiyang, Guizhou, China.

### 3.2. Materials

The 1D and 2D NMR spectra (tetramethylsilane as internal standard, MeOH-*d*_4_ as solvent) were collected with a nuclear magnetic resonance instrument (Bruker DPX 400). HR-ESI-MS data were collected by Thermo Fisher QE Focus (Waltham, MA, USA). Semi-preparative HPLC was used in the experiment, and the chromatographic column was Waters SunFireTM C_18_ (5 μm, 10 × 250 mm) from COSMOSIL, with an LC-6AD pump (Shimadzu, Kyoto, Japan) and RID-20A (reflective refractive index detector). Silica gel (200–300 mesh, Qingdao Haiyang Silica Gel Drying Agent Co., Ltd., Qingdao, China) was used for column chromatography, and ODS-gel (50 μm, YMC Group, Kyoto, Japan) was used for column chromatography purification. Pyrrolidine dithiocarbamate (PDTC), *α*-glucosidase, ascorbic acid, ABTS, and DPPH were purchased from Solarbio (Beijing, China). The IL-6 ELISA kit and TNF-*α* ELISA kit were purchased from Elite Biotechnology Co., Ltd. (Wuhan, China). The RAW264.7 cell line was purchased from ATCC (Manassas, VA, USA). The biological activity was evaluated using DMEM (Gibicio, Waltham, MA, USA), fetal bovine serum (Biológica, Argentina, Natocor Industry, Florencio Varela, Argentina), lipopolysaccharides (Sigma-Aldrich, Burlington, MA, USA), an MTT cell proliferation and cytotoxicity assay kit (Sigma-Aldrich, Burlington, MA, USA), and a nitric oxide (NO) assay kit (Sigma-Aldrich, Burlington, MA, USA).

### 3.3. Extraction and Isolation

The air-dried roots of *R. roxburghii* (40 kg) were soaked in MeOH (3 times × 20 L). After the organic solvent in the extract was removed by vacuum concentration, the crude extract (4 kg) was obtained. The crude extract was eluted by gradient elution with D101-type macroporous adsorption resin (MeOH-H_2_O = 3:7–1:0). A 30% methanol–water partial extract (408 g) and a 70% methanol–water partial extract (300 g) were obtained. The 30% methanol–water extract was eluted with a silica gel (200–300 mesh) column chromatographic gradient (CH_2_Cl_2_-CH_3_OH = 1:0, 20:1, 15:1, 10:1, 7:1; 3:1, 0:1) to obtain seven fractions (Fr.A–Fr.J). Fraction C (43.0 g) was separated by ODS column chromatography and MeOH-H_2_O (1:9–1:0) to obtain fractions C1–C8. The fractions C4 (720 mg) and C6 (840 mg) were eluted by semi-preparative HPLC with isocratic elution (MeOH-H_2_O, 51:49, 38:62, flow rate: 3 mL·min^−1^) to create compounds **1** (34.5 mg, *tR* 12.9 min), **4** (37 mg, *tR* 28.4 min), and **5** (44 mg, *tR* 33.5 min). The 70% methanol–water extract was eluted with a silica gel (200–300 mesh) column chromatographic gradient (CH_2_Cl_2_-CH_3_OH = 1:0, 30:1, 20:1, 15:1, 10:1, 7:1; 3:1, 0:1) to obtain 9 main fractions (Fr.A–Fr.I). Fraction A was obtained by using a silica gel column (200–300 mesh) with CH_2_Cl_2_-CH_3_OH gradient elution (1:0, 50:1, 30:1, 20:1, 15:1, 10:1, 7:1; 3:1, 0:1) and subjected to ODS column chromatography and MeOH-H_2_O (1:9–1:0) to obtain factions A1–A7. Factions A2 (630 mg) and A6 (610 mg) were subjected to semi-preparative HPLC (MeOH-H_2_O, 50:50 and 51:49, flow rate: 3 mL·min^−1^) to produce compounds **2** (42 mg, *tR* 16.5 min), **3** (27 mg, *tR* 21.3 min), **6** (33 mg, *tR* 24.1 min), and **7** (30 mg, *tR* 29.5 min).

#### Characterization of Compound **1**

Roxbubenzoate A (**1**): pale-yellow oily substance; ${\left[\alpha \right]}_{D}^{20}$ = 36.25 (*c* = 0.32, MeOH) (Figure S8), UV (MeOH) *λ*_max_ 216, 277 nm, IR (KBr)*ν*_max_ 3420, 1720, 1337, 1229, 1116, 1047 cm^−1^. HRESIMS: *m*/*z* 485.1266 [M + Na]^+^ (calcd. for C_19_H_26_O_13_Na^+^ 485.1265). CD: (c = 0.4 mg/mL, MeOH) 208 nm (Δ*ε* + 1.57) and 275 nm (Δ*ε* + 0.28), a negative Cotton effect at 231 nm (Δ*ε* − 0.38). ^1^H-NMR (methanol-*d*_4_, 400 MHz) and ^13^C-NMR (methanol-*d_4_*, 100 MHz) are shown in Table 1.

### 3.4. RAW264.7 Cell Culture

RAW264.7 cells were cultured in DMEM containing 1% penicillin/streptomycin and 10% bovine fetal serum in an incubator (5% CO_2_, 37 °C).

### 3.5. RAW264.7 Cell Viability Experiment

RAW264.7 cells in the logarithmic growth phase were taken and adjusted to 1.5 × 10^5^ cells/mL. The cells were inoculated in 96-well plates and divided into a control group, a blank group, and an experimental group with different concentrations. Each group had 4 duplicate wells and was incubated for 24 h (37 °C). Different concentrations of compounds (100 μL) were added to 96-well plates, and 100 μL of culture medium was added to the blank group and the control group, respectively, and incubated for 24 h. MTT (5 mg/mL, 10 μL) was incubated for 4 h (37 °C) per well. The OD value was measured at 450 nm using a microplate reader to calculate the cell viability.

### 3.6. Assay of Inflammatory Factor Inhibition

RAW264.7 cells were added to a 96-well plate (100 μL/well) at 1.5 × 10^5^ cells/mL and incubated for 24 h (37 °C). The control group, model group, and experimental group were set up, with 4 replicates in each group. After incubation, the supernatant was discarded. The cells in the experimental group were treated with different concentrations of compounds, and each group was incubated with lipopolysaccharides (2 μg/mL) for 24 h (37 °C). According to the steps and requirements of the kit instructions, NO, TNF-*α*, and IL-6 content was detected.

### 3.7. α-Glucosidase Inhibition Assay

For this assay, 20 μL of each sample, 20 μL of *α*-glucosidase solution (0.6 U/mL), and PBS solution (20 μL) were mixed, added to a 96-well plate, and incubated at 37 °C for 10 min, and then, pNPG solution (20 μL) was added and incubation continued for 30 min. At the end of incubation, the reaction was terminated with the addition of 80 μL of NaCO_3_ (0.2 M) [6]. The release of p-nitrophenol was measured by placing the 96-well plate at 405 nm on an enzyme meter (positive control: acarbose). The test was repeated three times for all samples.

Inhibition (%) = (1 − A_405 sample_/A_405 control_) × 100%.

### 3.8. DPPH and ABTS Free Radical-Scavenging Assays

In order to explore the antioxidant capacity of compounds **1**–**7**, DPPH and ABTS free radical-scavenging methods were used in this experiment (ascorbic acid as a positive control). During the experimental operation, the antioxidant test method proposed by Zhu et al. [24] was used as a reference. Different concentrations of each compound solution were added to 96-well plates, and then 100 μL of DPPH/ABTS solution was added to each well, and the reaction was performed at room temperature for 30 min (DPPH)/6 min (ABTS). After the reaction, the OD values of each well were measured at 517 nm (DPPH)/734 nm (ABTS) using a microplate reader. Finally, according to the measured OD value, the DPPH and ABTS free radical-scavenging rates of each sample were calculated. In order to ensure the accuracy and reliability of the experimental data, all samples were tested three times.

Clearance = [1 − (A _sample group_ − A _control group_)/A _blank group_] × 100%

### 3.9. Network Pharmacological Studies

The structure of **3** was drawn with ChemDraw, and the SMILES of **3** were obtained through the Pubchem database (https://pubchem.ncbi.nlm.nih.gov). Targets were filtered from the OMIM database (https://www.omim.org) and Genecards database (https://www.genecards.org); disease targets were filtered using a threshold of mean ≥ 1.08, and 1997 inflammatory targets were retained. In addition, a total of 158 constituent gene targets were obtained from Swiss Target Prediction (https://prediction.charite.de/), and 106 intersecting genes were obtained [25]. Microbial Informatics (http://www.bioinformatics.com.cn) was used to draw the Veeny diagram of **3** and common inflammation targets, and the String database (https://cn.string-db.org) was used to construct the protein interaction network (PPI) of cross-targets and save the TSV format file. The TSV file was imported into Cytoscape 3.10.0 software so that a visualization of the PPI network could be performed and the core targets could be filtered out [26].

The pathway enrichments of the KEGG (Kyoto Encyclopedia of Genes and Genomes) and GO (Gene Ontology) were obtained through the DAVID database (https://david.ncifcrf.gov/), and the top 20 pathways were selected to create bubble plots, which were drawn using Microbial Informatics (http://www.bioinformatics.com.cn) to facilitate visual analysis of the data [1].

### 3.10. Molecular Docking Verification

The 3D structure of **3** was obtained from ChemDraw 20.0 software. Receptor structures were searched for from the PDB Protein Structure Database while pre-processing the receptors. Docking was run through AutoDock Vina software (Version 1.2.3), which generates a binding energy for each docked conformation, with lower binding energies usually indicating tighter binding of the ligand to the receptor, and the binding energies were used to filter the possible effective binding conformations. In addition, the binding pattern of the ligand and receptor was demonstrated using the visualization software PyMOL (Version 2.4), and the interaction between the ligand and amino acid residues in the active site of the receptor could be observed in order to assess the plausibility and reliability of the docking results [27].

### 3.11. Statistical Analysis

In this study, all experiments were strictly repeated three times to ensure the reliability of the data. The experimental results are expressed as mean ± standard deviation (n = 3). Statistical analysis was performed using SPSS 26. software, with ^###^ *p* < 0.001, * p < 0.05, ** p < 0.01, and *** p < 0.001 showing statistical significance.

## 4. Conclusions

In summary, one previously undescribed compound (**1**) and six known compounds (**2**–**7**) were isolated from the roots of *R. roxburghii*. Roxbubenzoate A (**1**) possessed a benzoyl glucuronosyl glycerol scaffold featuring a rare *α*-glucuronosyl linkage. Compounds (**2**–**7**) were isolated from the roots of *R. roxburghii* for the first time. Compounds (**1**–**7**) were tested for their anti-inflammatory, *α*-glucosidase-inhibitory, and radical-scavenging activities. Compound **3** showed the greatest inhibitory effect on the release of NO in LPS-induced RAW264.7 macrophages, with an IC_50_ value of 7.8 ± 0.2 μM. It exhibited significant anti-inflammatory activity, and its mechanism was evaluated based on network pharmacology and biological verification. The results of the network pharmacology analysis and molecular docking showed that **3** may exert an anti-inflammatory effect by binding to TNF-*α* and IL-6 targets. In the ELISA experiment, compound **3** displayed the most active anti-inflammatory activity by targeting the crucial protein IL-6, indicating that **3** may be the main active ingredient for the anti-inflammatory effect of *R*. *roxburghii* roots. This work will greatly expand the chemical diversity and pharmacological prospects of *R. roxburghii* roots and provide important information for their development and utilization.

## Figures and Tables

**Table 1:** ^1^H and ^13^C NMR data of compound **1** (*δ* in ppm).

NO.	*δ*_C_	*δ*_H_ (*J* in Hz)
**1**	66.6	4.39 (1H, dd, *J* = 11.5, 4.2 Hz) 4.34 (1H, dd, *J* = 11.5, 4.8 Hz)
**2**	69.5	4.17 (H, m)
**3**	70.6	3.90 (1H, dd, *J* = 10.0, 5.2 Hz) 3.54 (1H, dd, *J* = 10.0, 6.6 Hz)
**1′**	101.2	4.89 (1H, d, *J* = 3.7 Hz)
**2′**	73.3	3.47 (1H, dd, *J* = 9.3, 3.4 Hz)
**3′**	74.4	3.68 (1H, t-like, *J* = 9.3 Hz)
**4′**	73.2	3.50 (1H, t-like, *J* = 9.9 Hz)
**5′**	73.1	4.08 (1H, d, *J* = 9.9 Hz)
**6′**	171.9	-
**1″**	121.2	-
**2″**	108.3	7.37 (1H, s)
**3″**	148.9	-
**4″**	142.0	-
**5″**	148.9	-
**6″**	108.3	7.37 (1H, s)
**7″**	167.9	-
**3″, 5″-OCH_3_**	56.9	3.90 (6H, s)
**6′-OCH_3_**	52.7	3.66 (3H, s)

Measured in CD_3_OD (^1^H: 400 MHz; ^13^C: 100 MHz).

## Data Availability

The data are contained within the article or Supplementary Materials.

## Supplementary Materials

The following Supporting Information can be downloaded at https://www.mdpi.com/article/10.3390/ijms26031353/s1.
