# Supplementary material for: Biological Activities of Constituents from Rosa roxburghii and Their Mechanisms Based on Network Pharmacology and Biological Verification
Source: Int J Mol Sci. 2025 Feb 5;26(3):1353. doi: 10.3390/ijms26031353 (PMC11818921; doi:10.3390/ijms26031353)
Supplement: Supplementary file 1 [file ijms-26-01353-s001.zip › ijms-3466609-supplementary.pdf]

Supporting Information

Biological activities of constituents from *Rosa roxburghii* and their mechanism based on network pharmacology and biological verification

Li-juan Xiang[1], Shuang Zhang[1], Ming-liang Luo[2], Xing-xiang Long[3], Ying Zhou[1,*] and Xin Yin[2,*]

College of Pharmacy, Guizhou University of Traditional Chinese Medicine, Guiyang 550025, P. R. China

Correspondence

Ying Zhou[1,*] (E-mail: zhouying@gzy.edu.cn) and **Xin Yin[2,*]** (E-mail: yinxin@gzy.edu.cn)

[1]Li-juan Xiang and [1]Shuang Zhang contributed equally to this work.

Legends for Tables and Figures

Table S1: ^{13}C-NMR (100 MHz) Data of compounds **2-7** in CD_3OD
Table S2: The effect of compounds (**1-6**) on the cell viability of RAW264.7 cells, and NO inhibition rate (Pyrrolidine dithiocarbamate (PDTC) is a positive control).
Figure S1: ^{1}H-NMR Spectrum of Compound **1**
Figure S2: ^{13}C-NMR spectrum of Compound **1**
Figure S3: DEPT-135 spectrum of Compound **1**
Figure S4: HSQC spectrum of Compound **1**
Figure S5: HMBC spectrum of Compound **1**
Figure S6: ^{1}H-^{1}H COSY spectrum of Compound **1**
Figure S7: HR-ESI-MS spectrum of Compound **1**
Figure S8: ORD spectrum of Compound **1**
Figure S9: CD spectrum of Compound **1**
Figure S10: UV spectrum of Compound **1**
Figure S11: IR spectrum of Compound **1**
Figure S12: ^{1}H-NMR Spectrum of Compound **2**
Figure S13: ^{13}C NMR spectrum of Compound **2**
Figure S14: ^{1}H-NMR Spectrum of Compound **3**
Figure S15: ^{13}C NMR spectrum of Compound **3**
Figure S16: ^{1}H-NMR Spectrum of Compound **4**
Figure S17: ^{13}C NMR spectrum of Compound **4**
Figure S18: ^{1}H-NMR Spectrum of Compound **5**
Figure S19: ^{13}C NMR spectrum of Compound **5**
Figure S20: ^{1}H-NMR Spectrum of Compound **6**
Figure S21: ^{13}C NMR spectrum of Compound **6**
Figure S22: ^{1}H-NMR Spectrum of Compound **7**
Figure S23: ^{13}C NMR spectrum of Compound **7**

Table S1. ^{13}C-NMR (100 MHz) Data of compounds **2-7** in CD_3OD

**NO.**	**2**	**3**	**4**	**5**	**6**	**7**
**1**			42.3	38.8	130.2	121.9
**2**	80.5	84.9	160.3	49.5	133.2	132.7
**3**	44.1	73.6	126.0	65.7	116.2	116.3
**4**	197.7	198.2	188.6	43.0	156.6	164.1
**5**	165.4	165.3	132.4	125.4	116.2	116.3
**6**	103.3	96.3	165.9	138.2	133.2	132.7
**7**	168.5	169.7	27.3	20.0	30.0	168.8
**8**	96.2	96.3	36.2	30.3	46.3	
**9**	164.8	164.5	77.7	69.2	211.5	
**10**	103.3	101.7	21.9	23.3	30.0	
**11**			11.6	28.9		
**12**			26.0	28.8		
**13**			25.9			
**1′**	131.8	128.7	104.3			
**2′**	114.7	129.3	75.4			
**3′**	146.9	115.6	78.4			
**4′**	146.5	159.1	77.7			
**5′**	116.2	115.8	77.9			
**6′**	119.2	129.3	62.8			

Table S2. The effect of compounds (**1-6**) on the cell viability of RAW264.7 cells, and NO inhibition rate (Pyrrolidine dithiocarbamate (PDTC) is a positive control).

Sample number	Concentration (μM)	Cell viability (%)	NO inhibition rate (%)
**1**	50	100.0%	0.0%
**2**	50	100.0%	31.3%
**3**	50	100.0%	92.5%
**4**	50	98.8%	6.6%
**5**	50	98.3%	0.8%
**6**	50	98.1%	0.8%
DPTC	5	98.5%	69.1%
LPS (2μg/mL)	/	100.0%	0.0%
Normal group	/	100.0%	100.0%

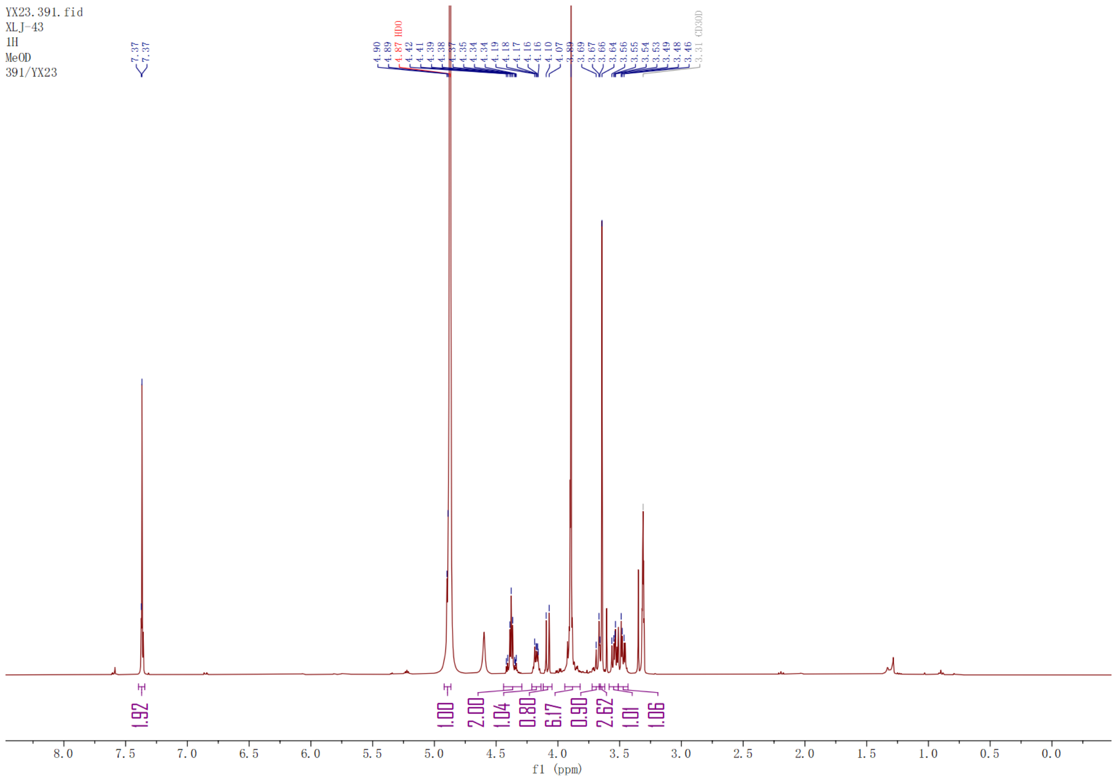

Figure S1. ^{1}H-NMR Spectrum of Compound **1**

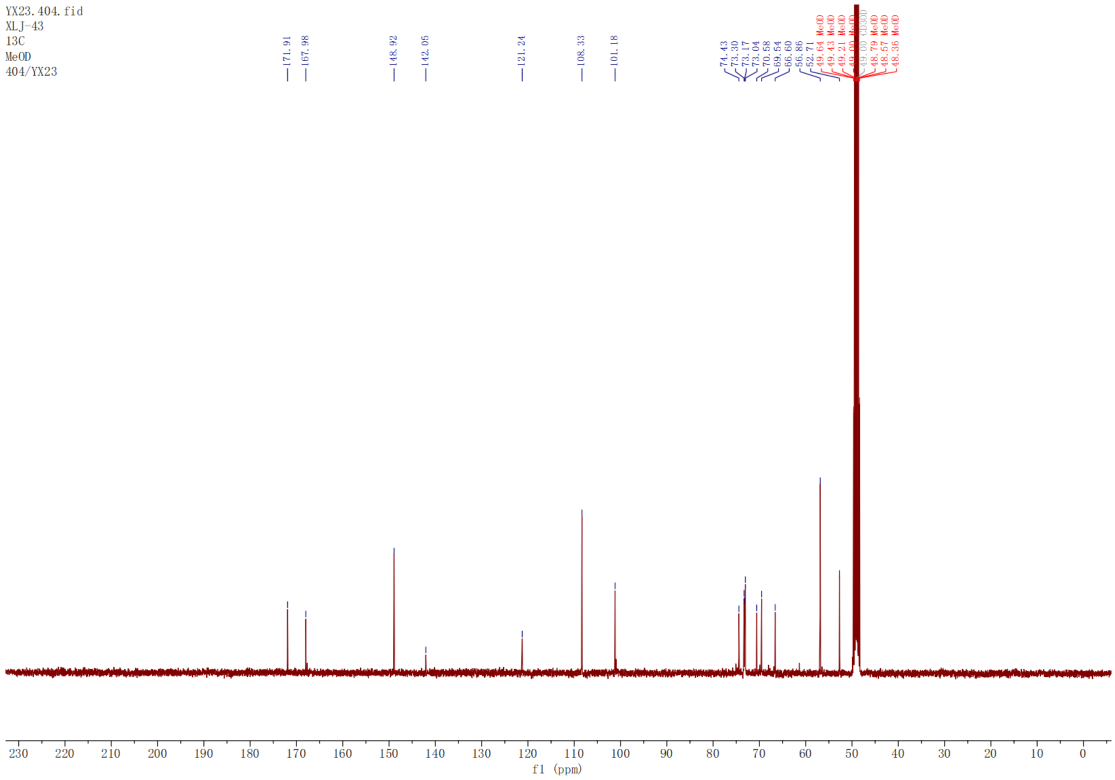

Figure S2. ^{1}C-NMR Spectrum of Compound **1**

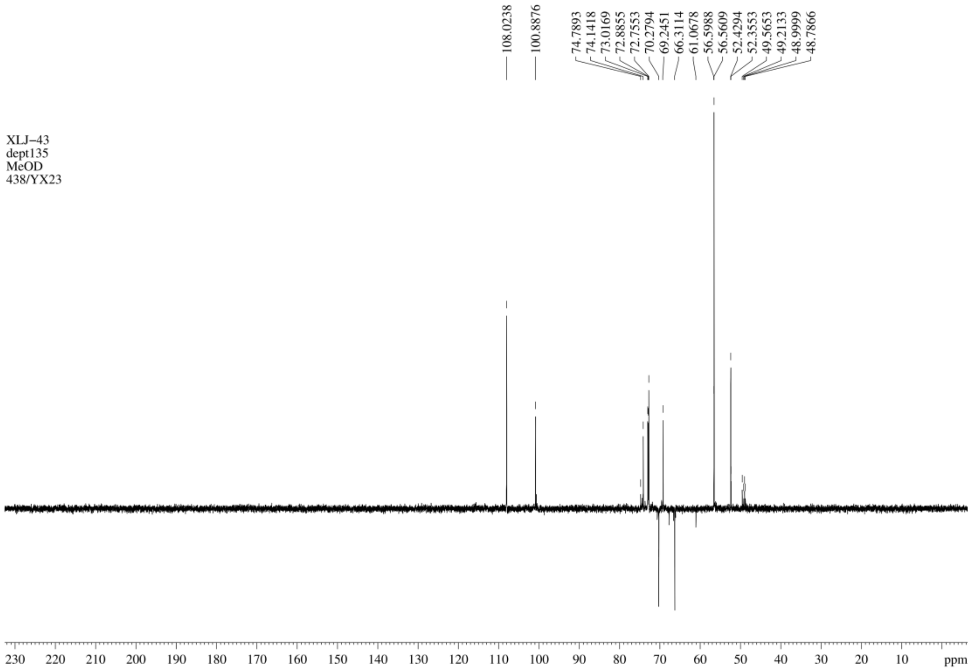

Figure S3. DEPT-135 spectrum of Compound **1**

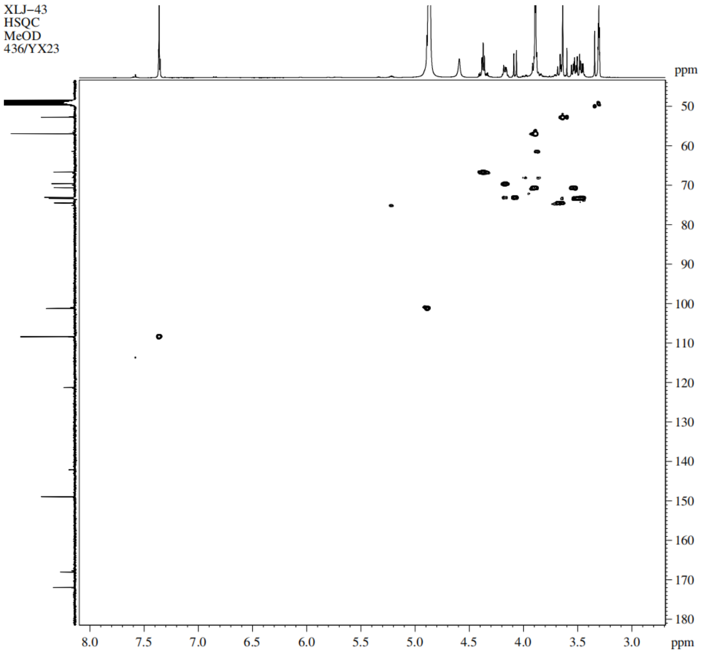

Figure S4. HSQC spectrum of Compound **1**

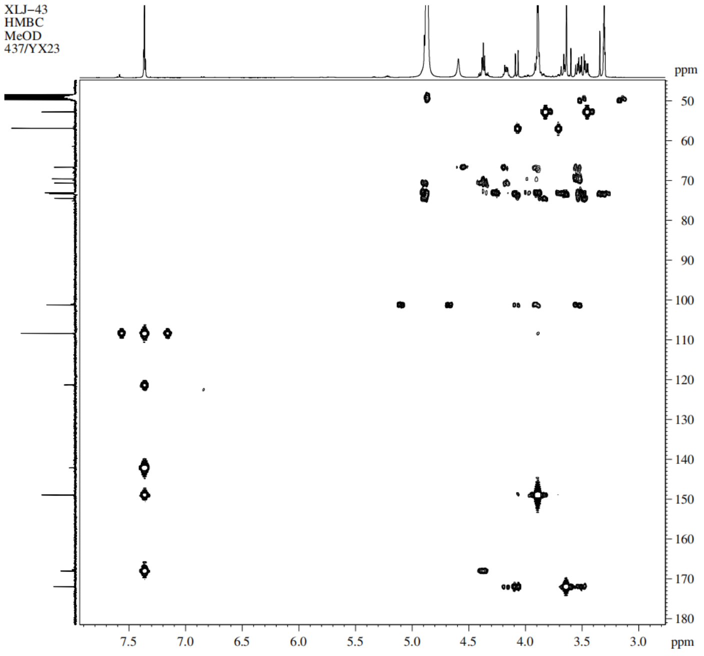

Figure S5. HMBC spectrum of Compound **1**

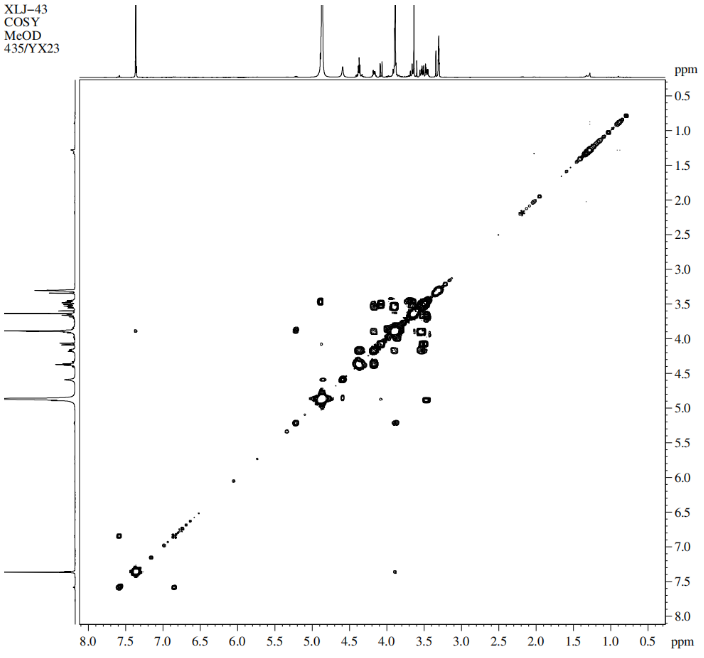

Figure S6. ^{1}H-^{1}H COSY spectrum of Compound **1**

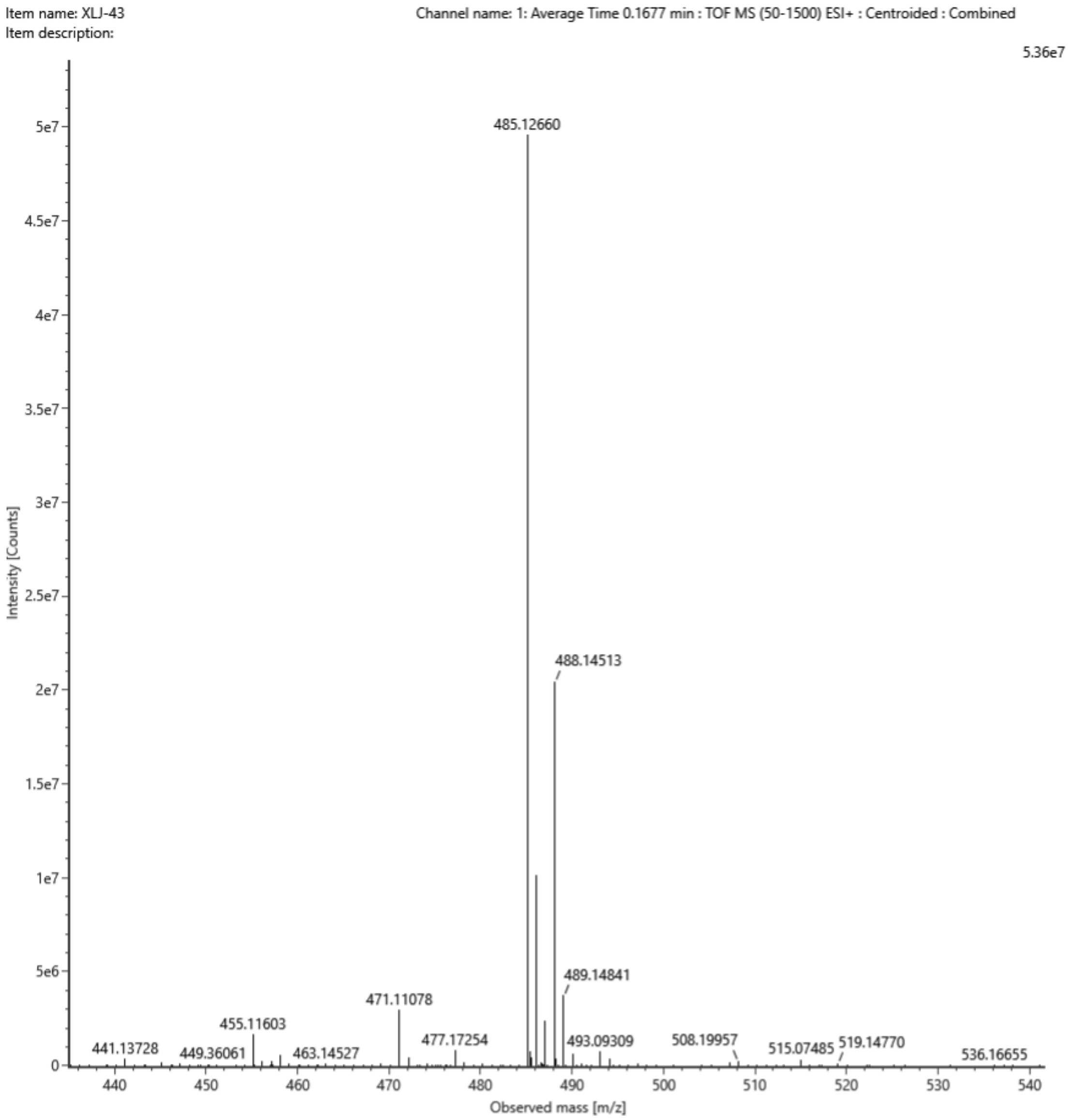

Figure S7. HR-ESI-MS spectrum of Compound **1**

Rudolph Research Analytical

This sample was measured on an Autopol VI, Serial #91058
Manufactured by Rudolph Research Analytical, Hackettstown, NJ, USA.

Measurement Date : Wednesday, 30-OCT-2024

Set Temperature : 20.0

Time Delay : Disabled

Delay between Measurement : Disabled

n	Average	Std.Dev.	% RSD	Maximum	Minimum
5	36.25	0.00	0.00	36.25	36.25

S.No	Sample ID	Time	Result	Scale	OR °Arc	WLG.nm	Lg.mm	Conc.g/100ml	Temp.
1	XLJ-43	03:48:21 PM	36.25	SR	0.116	589	100.00	0.320	20.0
2	XLJ-43	03:48:27 PM	36.25	SR	0.116	589	100.00	0.320	20.0
3	XLJ-43	03:48:34 PM	36.25	SR	0.116	589	100.00	0.320	20.0
4	XLJ-43	03:48:41 PM	36.25	SR	0.116	589	100.00	0.320	20.0
5	XLJ-43	03:48:48 PM	36.25	SR	0.116	589	100.00	0.320	20.0

Figure S8. ORD spectrum of Compound **1**

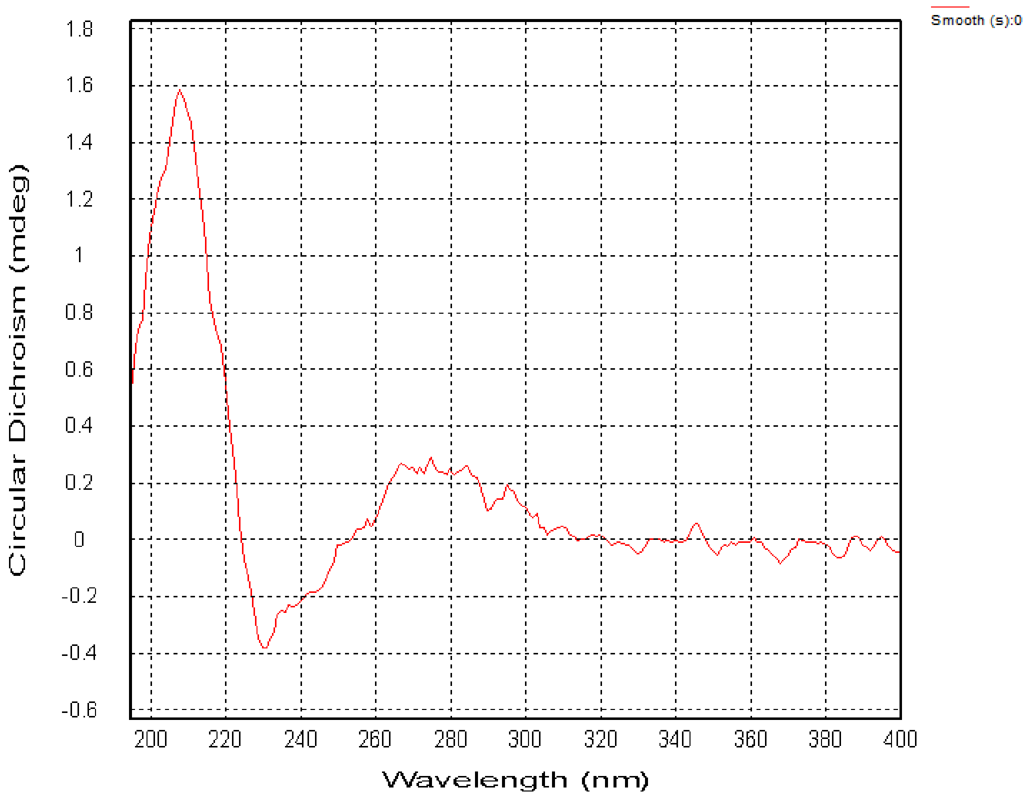

Figure S9. CD spectrum of Compound **1**

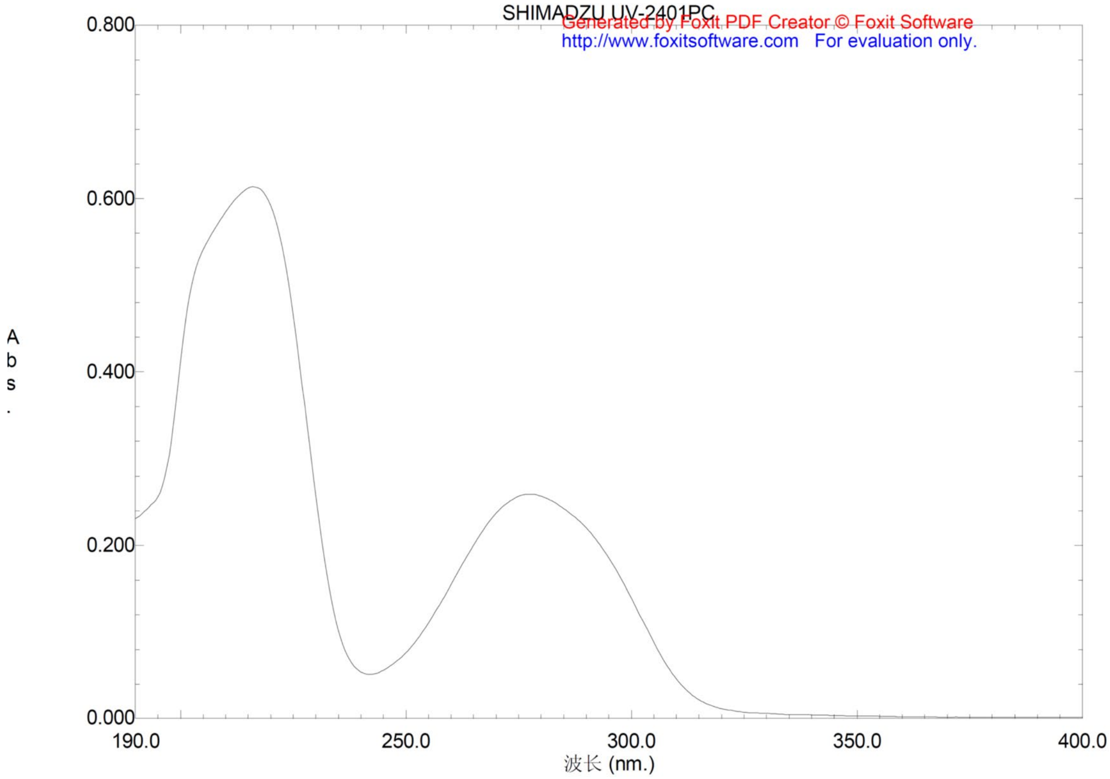

Figure S10. UV spectrum of Compound **1**

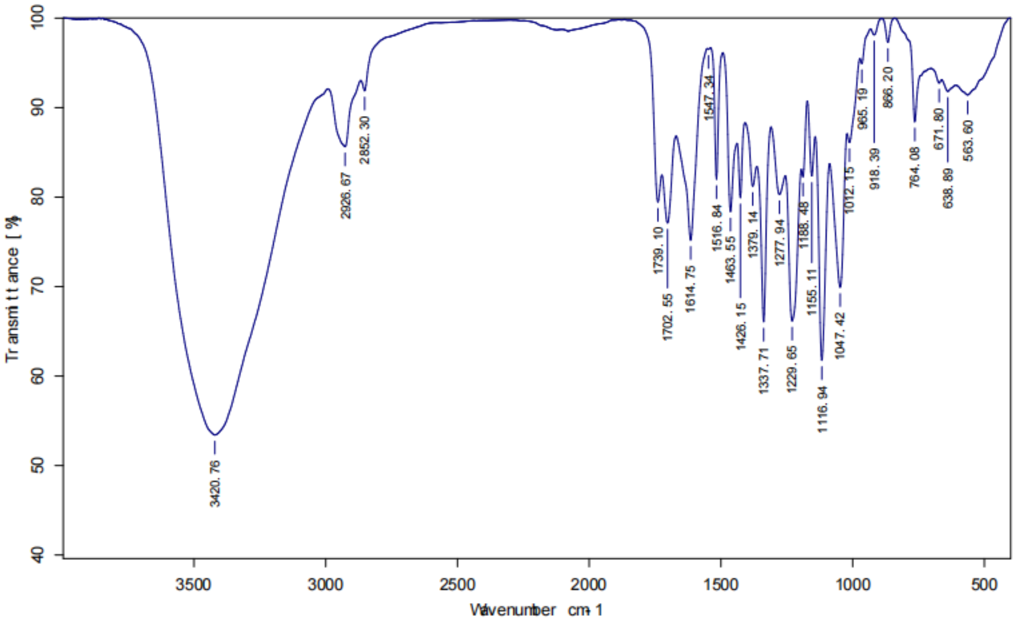

Figure S11. IR spectrum of Compound **1**

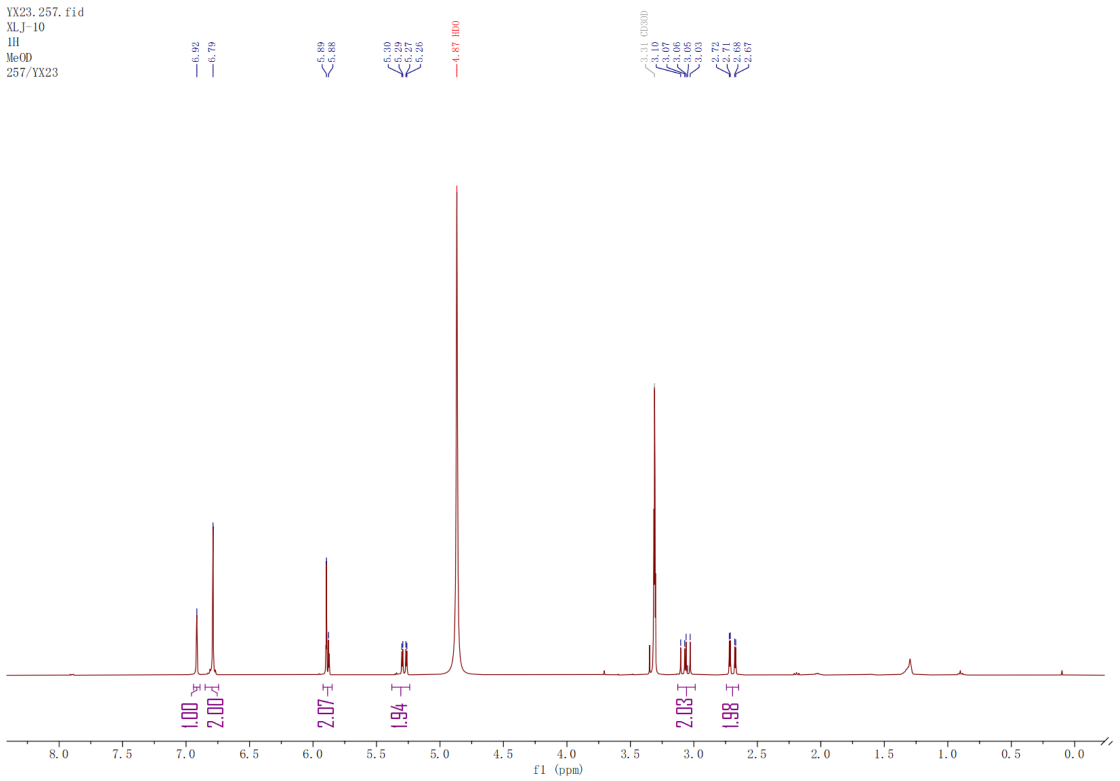

Figure S12. ^{1}H-NMR Spectrum of Compound **2**

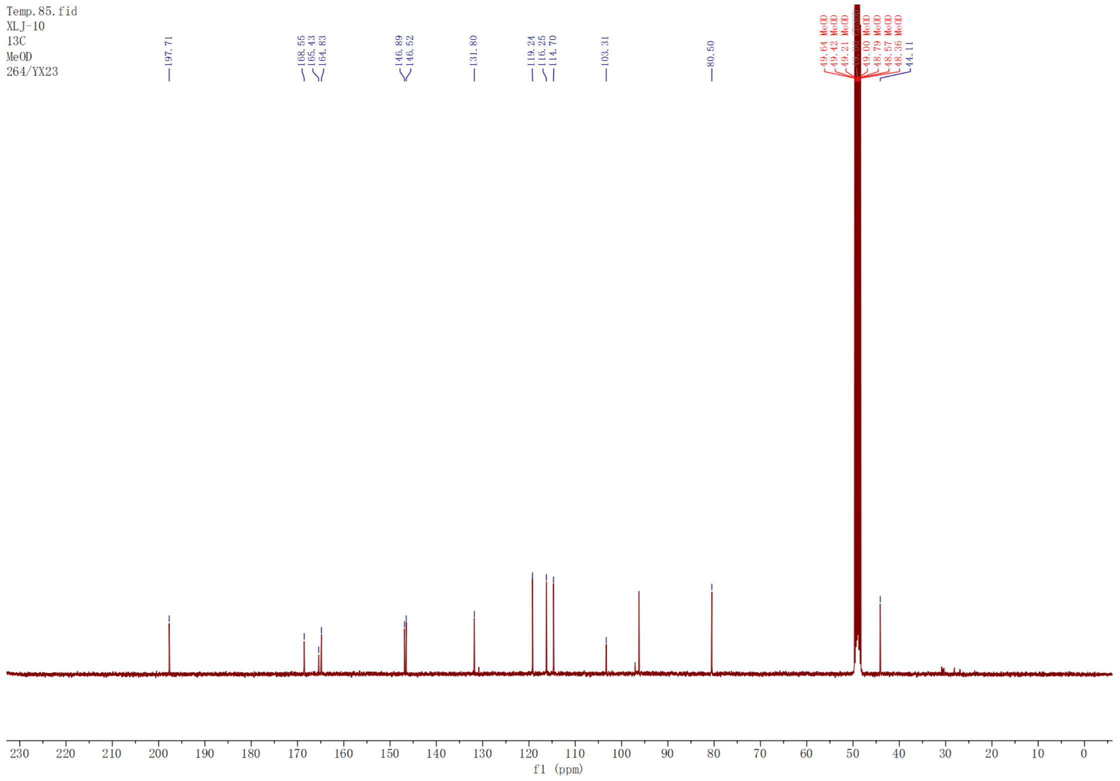

Figure S13. ^{13}C NMR spectrum of Compound **2**

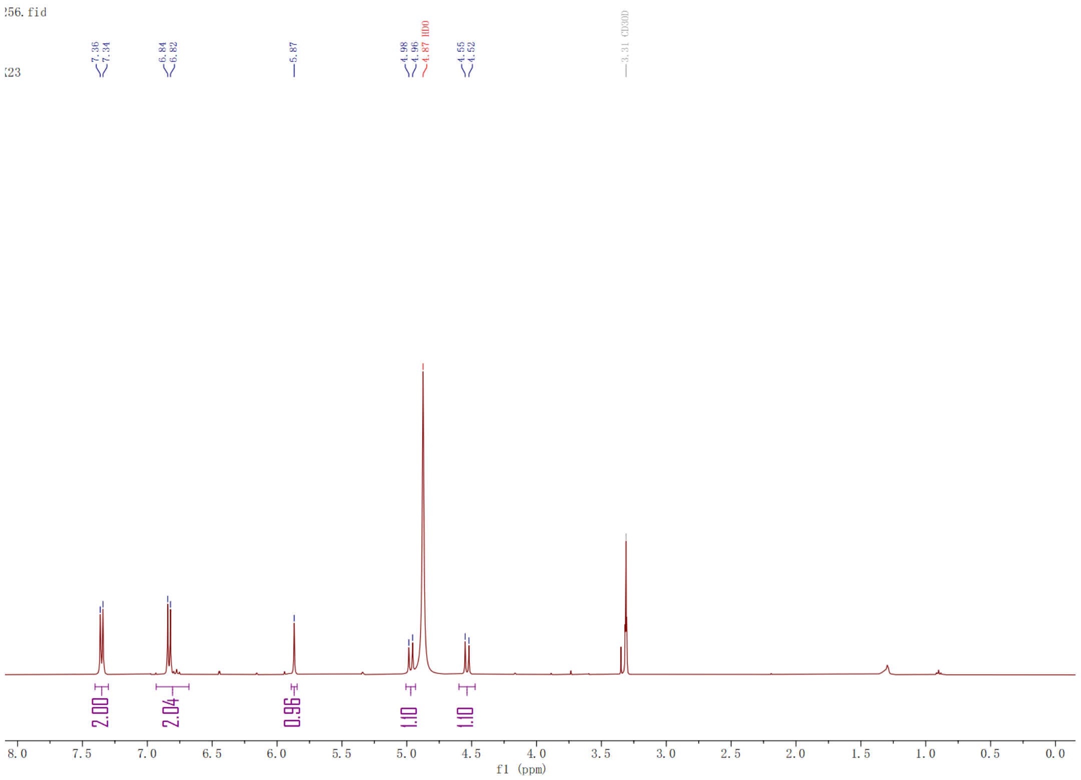

Figure S14. ^{1}H-NMR Spectrum of Compound **3**

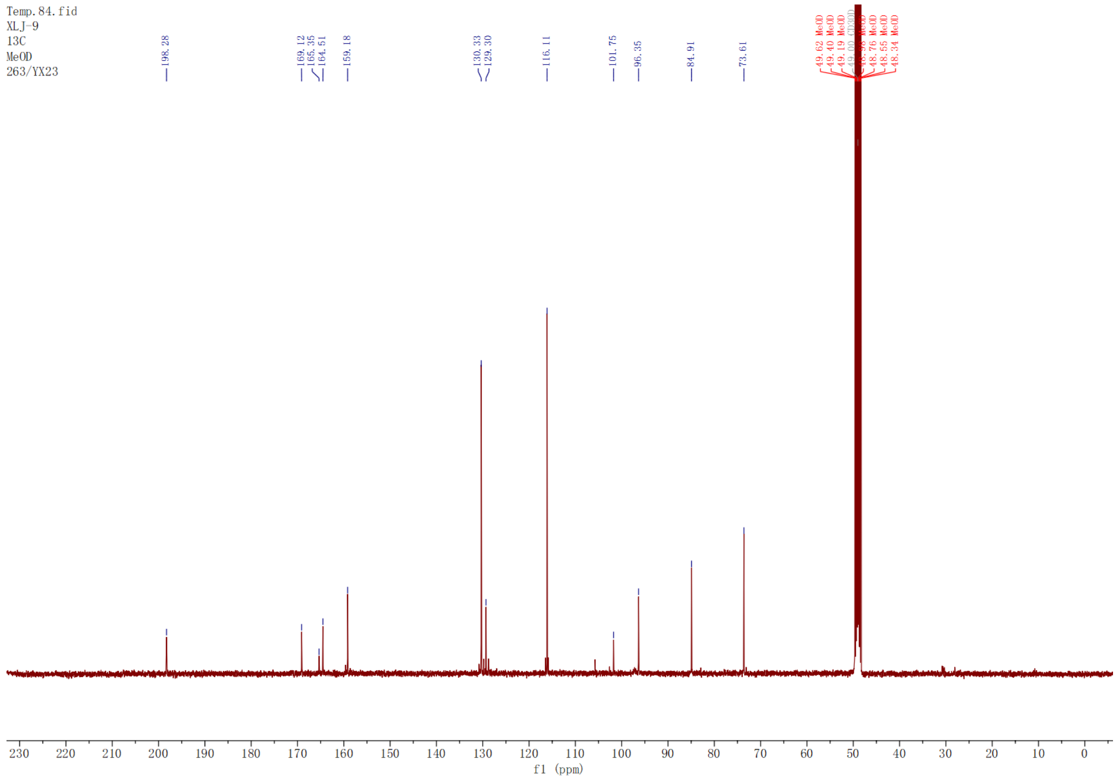

Figure S15. ^{13}C NMR spectrum of Compound **3**

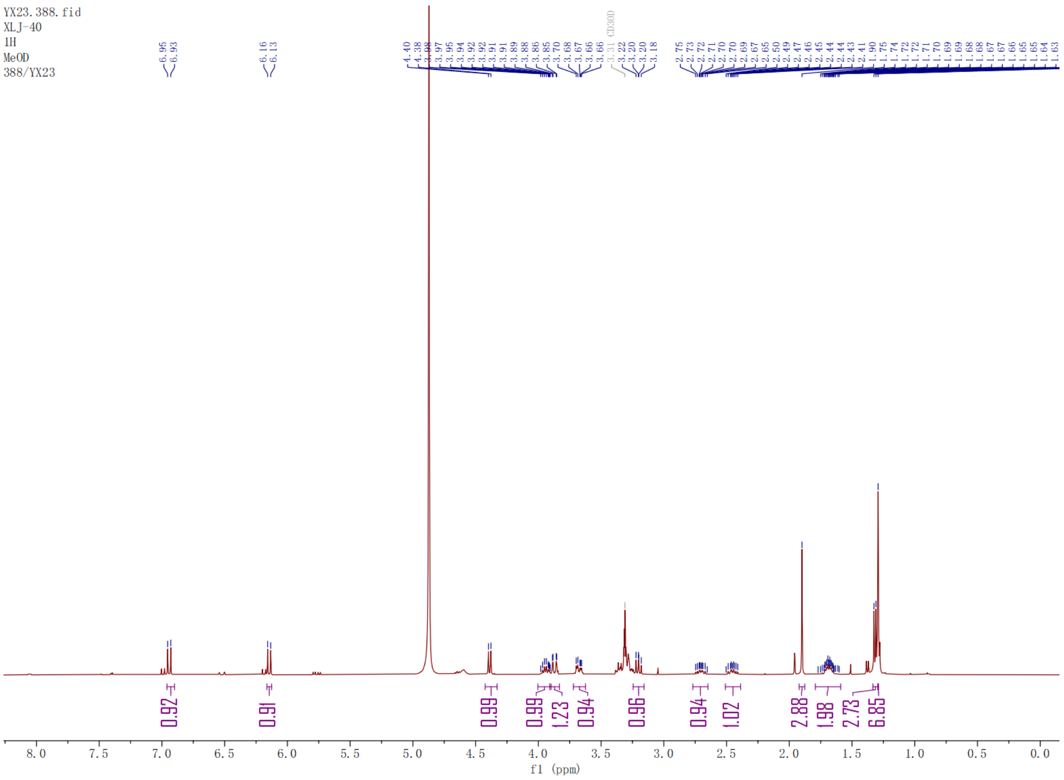

Figure S16. ^{1}H-NMR Spectrum of Compound **4**

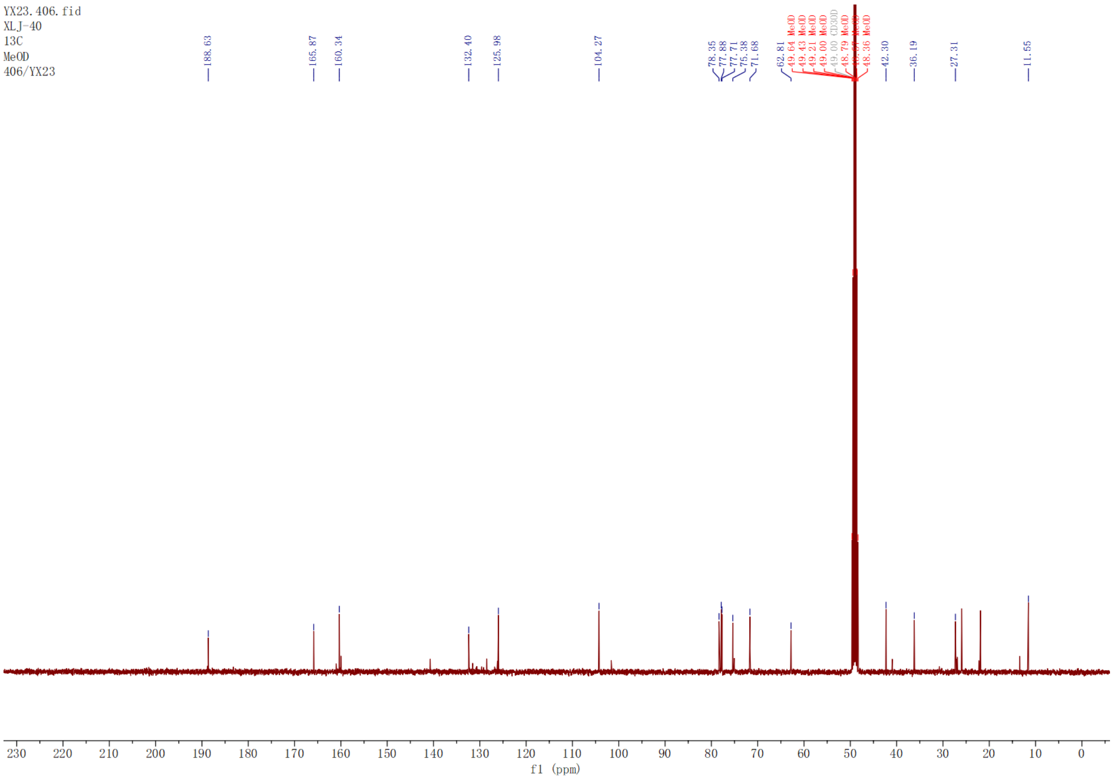

Figure S17. ^{13}C NMR spectrum of Compound **4**

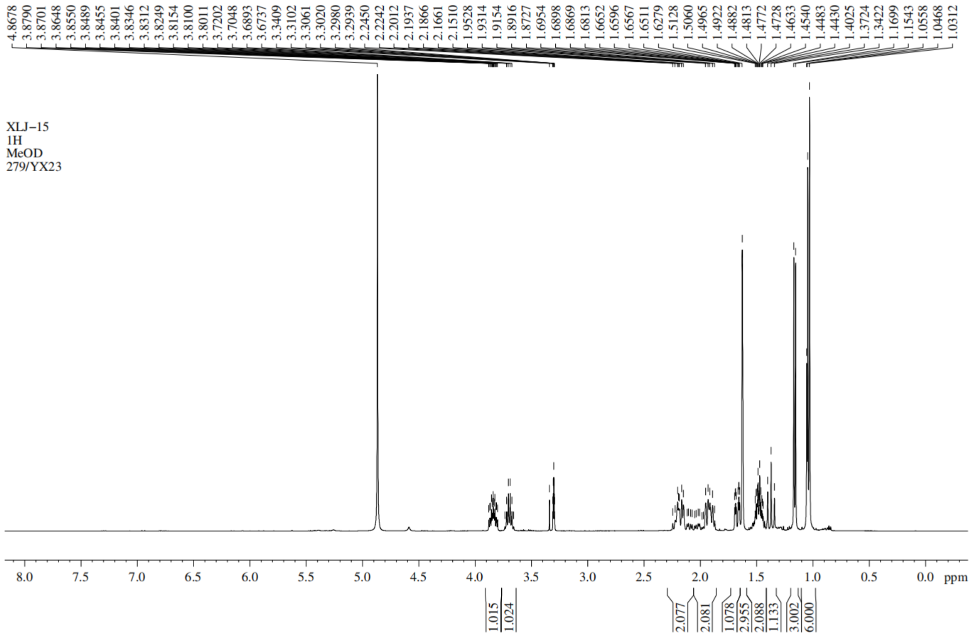

Figure S18. ^{1}H-NMR Spectrum of Compound **5**

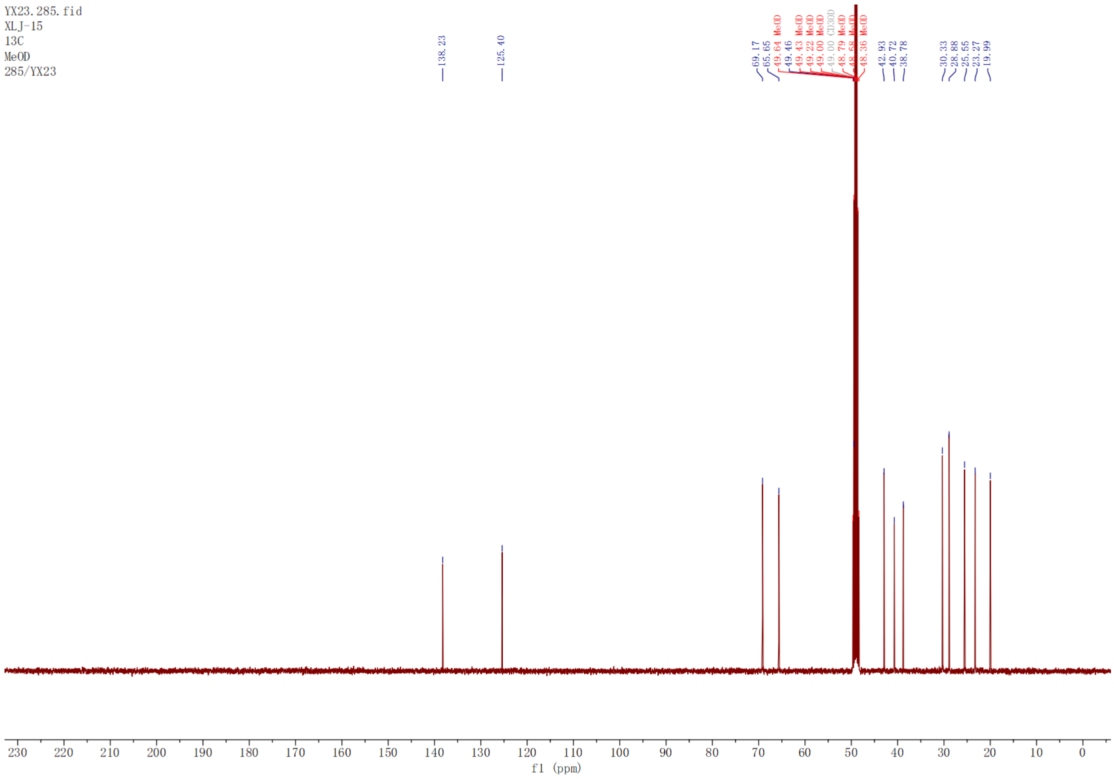

Figure S19. ^{13}C NMR spectrum of Compound **5**

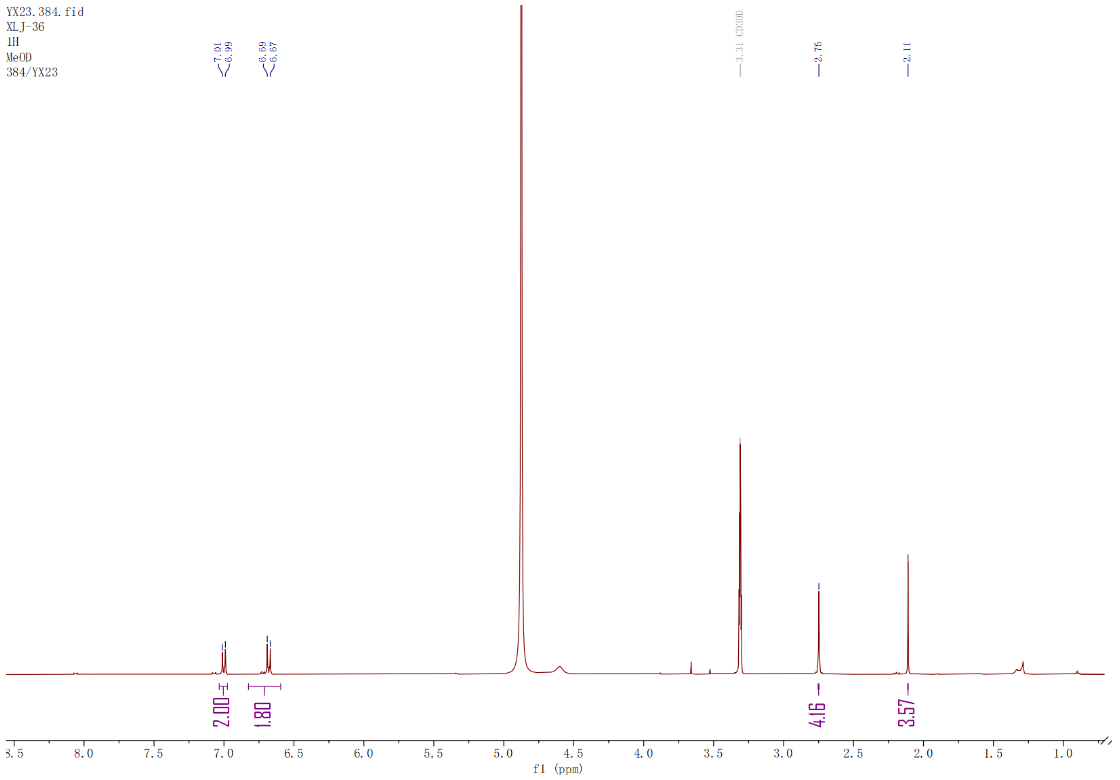

Figure S20. ^{1}H-NMR Spectrum of Compound **6**

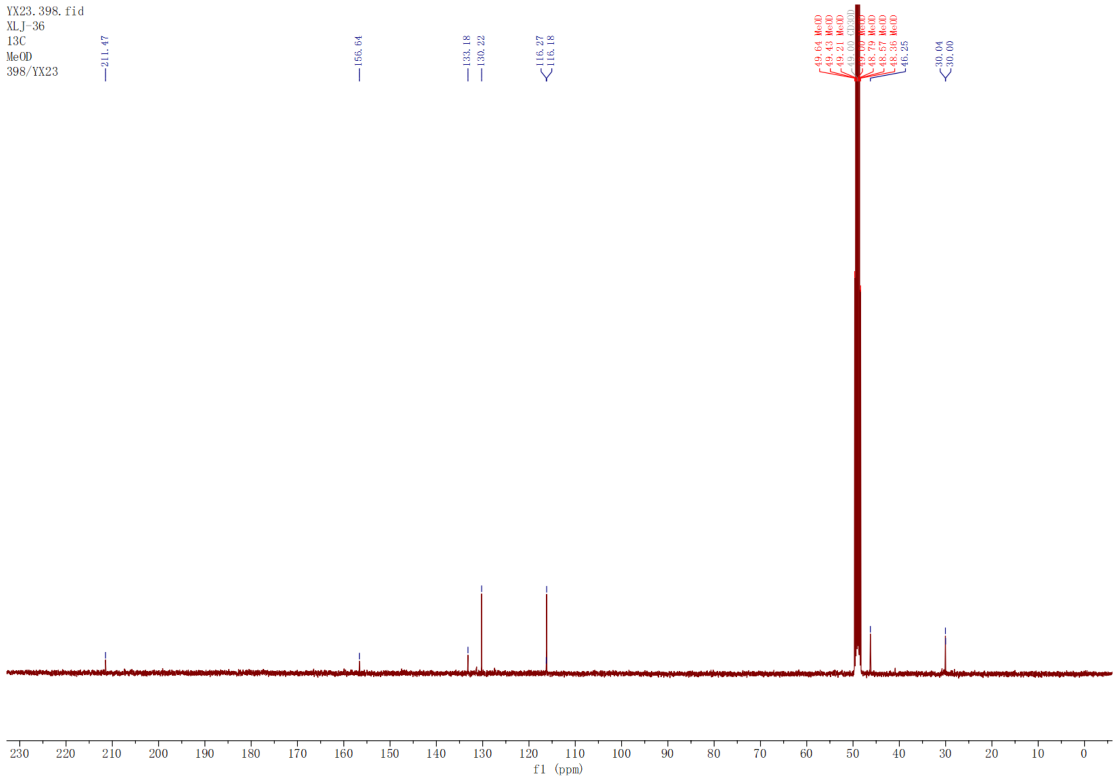

Figure S21. ^{13}C NMR spectrum of Compound **6**

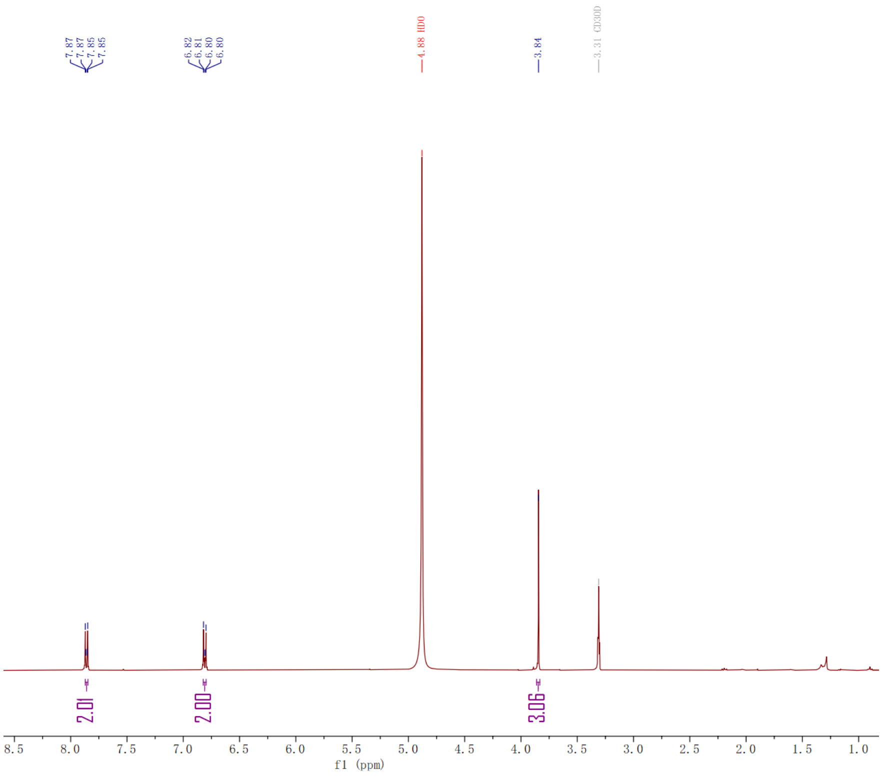

Figure S22. ^{1}H-NMR Spectrum of Compound **7**

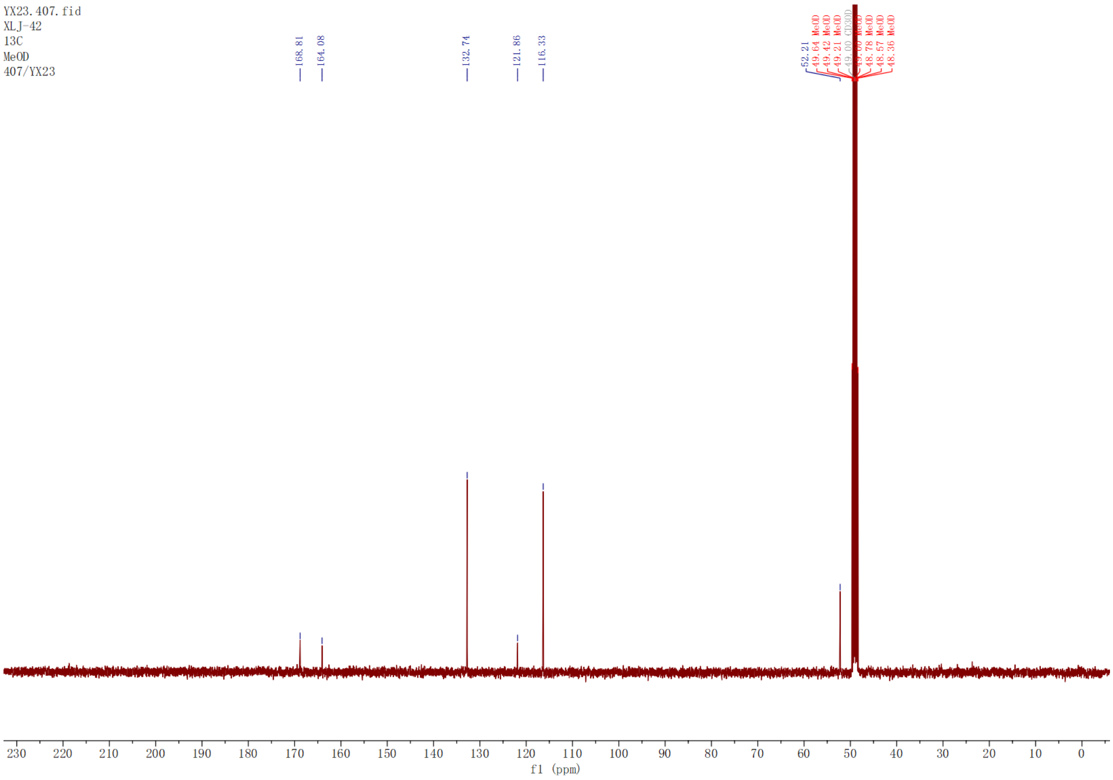

Figure S23. ^{13}C NMR spectrum of Compound **7**
